# “Suck It Up, Buttercup”: Understanding and Overcoming Gender Disparities in Policing

**DOI:** 10.3390/ijerph18147627

**Published:** 2021-07-18

**Authors:** Andréanne Angehrn, Amber J. Fletcher, R. Nicholas Carleton

**Affiliations:** 1 Département de Psychologie, Université du Québec à Trois-Rivières, Trois-Rivières, QC G8Z 4M3, Canada; 2 Department of Sociology and Social Studies, University of Regina, Regina, SK S4S 0A2, Canada; amber.fletcher@uregina.ca; 3 Department of Psychology, University of Regina, Regina, SK S4S 0A2, Canada; nick.carleton@uregina.ca

**Keywords:** police, gender, hegemonic masculinity, mental health

## Abstract

Women police officers report elevated symptoms of mental disorders when compared to men police officers. Researchers have indicated that the occupational experience of policing differs greatly among men and women. Indeed, police culture is characterized by hegemonic masculinity, which appears to negatively impact both men and women. The current study examined the contrast between the experiences of men and women police officers. Police officers (*n* = 17; 9 women) in Saskatchewan participated in semi-structured interviews. Thematic network analysis identified themes related to the experience of policing for both men and women police officers. There were six organizing themes identified in relation to the global theme of Gendered Experiences: (1) Discrimination; (2) Sexual Harassment; (3) Motherhood and Parental Leave; (4) Identity; (5) Stereotypically Feminine Attributes; and (6) Hegemonic Masculinity. Pervasive gender norms appear detrimental for both men and women police officers, as well as the communities they serve. The current results, coupled with the emerging disposition for progress expressed by police services, offer opportunities to develop tailored and focused interventions and policies to support police officers.

## 1. Introduction

Police officers appear to be at higher risk of several different mental disorders than the general population, arguably because of the nature of their work [1,2,3,4]. Women in Canadian municipal or provincial police organizations appear to be 1.66 times more likely than their men colleagues to screen positive for a mental disorder and report elevated symptoms of various mental disorders relative to men [3,5,6]. Furthermore, women in the police service report organizational stressors specific to gender (e.g., experiences of criticism, gender bias, discrimination, and sexual harassment) [7,8,9]. Accordingly, women and men police officers may have very different experiences of work-related stress, despite having the same occupation. Throughout this paper, we employ the language of gender, rather than sex, to describe participants’ identities. We acknowledge that terms like “male” and “female” are more commonly used to describe officers (i.e., “female police officer”), and we acknowledge the strong interconnection between gender and sex systems. However, the phenomena discussed in this paper are more strongly connected to gender than to sex. Therefore, gendered terms (e.g., “men officers”) are used. An increasing number of women are choosing to pursue careers in policing [10], which underscores the importance of understanding the potential gender differences that contribute to the mental health vulnerability associated with policing.

Workplace environment stressors for police (e.g., workload, work–life imbalance, challenges with communication) have been identified as negatively impacting mental health, even after considering the impacts of potentially psychologically traumatic events (PPTEs) [1,11,12,13]. Increased job-related stress and pressure, as well as reduced organizational support, appear to increase the risk for posttraumatic stress disorder among police officers [1,14]. Non-supportive work environments, in which officers experience discrimination, difficult peer relationships, or lack of clarity regarding their roles, have all been associated with poorer mental health outcomes [1,13,15]. 

Women police officers report greater organizational stress and greater associated distress (e.g., psychological and physiological symptoms, such as fear and sadness, associated with stressors) [16] than men [17]. Gender-related work stressors are among the most salient and distressing for women (e.g., perceptions of sexually offensive behaviors, vulgar/offensive language) [18,19]. Women police officers report experiencing greater bias, sexual and racial harassment, and underestimation of their physical abilities, as well as feeling less influential than their men colleagues [20]. For men police officers, the perception of having less influence on their work environment is subjectively more distressing while, in contrast, women police officers report bias, harassment, and token status as contributing more to their stress [20]. 

Overall, nearly half of women police officers report discrimination and prejudice based on their gender (i.e., 43 percent vs. 3 percent men uniformed officers, and 50 percent vs. 2 percent men detective officers) [21,22]. Over 40 percent of women police officers report believing that their gender in the context of policing directly contributes to their risk of negative health outcomes [23]. The above-mentioned report of gendered challenges for police women is consistent with other men-dominated occupations (e.g., military, construction, firefighters) in which women experience greater bias, discrimination, and harassment, as well as lower psychological wellbeing [24,25]. The nature of police work may also impact the extent to which women feel they can balance work and home demands. Women police officers report that police agencies lack adequate family-friendly policies, and many law enforcement agencies report that women police officers leave once they start a family [8,26]. 

For most women police officers, routine stressors intersect with gender-based discrimination and harassment as a result of police culture [27]. Police culture refers to the core belief system, norms, behaviors, and attitudes involved in policing. Core values of police culture can include conservatism, machismo, suspicion, isolation, and a focus on work and mission [28]. Police culture can favor an “us versus them” mentality, with an emphasis on solidarity with fellow officers; however, because an important subdomain of police culture is masculinity, women police officers may be perceived as outsiders, not fitting with the old-school officer ideal-type [29,30]. 

An important theoretical explanation for the gender inequalities in policing is hegemonic masculinity, or the embodiment of the idealized version of manhood, which influences the normative power held by men [31,32]. Beyond policing, researchers have focused on the dominance of men and the value of masculinity in order to better understand gender power dynamics within organizations [33,34]. Through hegemonic masculinity, masculinity is influenced in part by ethnicity, sexual orientation, and class [33,34]. Exerting masculinity can be perceived as integral to the work of policing, with men police officers being idealized as brave, rational, physically strong, and objective, with a general perception that women are less likely to fit this ideal stereotype [35]. Extreme hegemonic masculinity is valued in policing, wherein men may represent their virility through physical strength, sexual and misogynistic regard for women, and emphasizing interests designated as masculine (e.g., sports and hunting); in contrast, women police officers are often perceived as subordinate, with their authority over men not readily accepted or taken seriously despite their rank or authority [36,37]. 

The aforementioned difficulties faced by women police officers appear to provide possible explanations for at least some of the gender differences in mental disorder prevalence among police. The current study was designed to explore the experiences of policing from a gender perspective, in order to better understand how this may relate to police officers’ wellbeing [6]. Substantial media attention, but relatively little empirical research, has focused on the experiences and struggles of women police officers. The current study was designed to identify common and differing themes in the experiences of men and women police officers.

## 2. Materials and Methods

### 2.1. Recruitment and Procedures

The current study objectives were approved by the Saskatchewan Association of Chiefs of Police (SACP) after a brief proposal. The SACP sent a recruitment poster and a brief memo outlining participation in the study to police services across Saskatchewan. Interested participants independently contacted the researcher based on the research request distributed to the participating police services. Participants engaged in interviews in person or over the phone in a closed office. Ethical approval was received from the Research Ethics Board at the University of Regina and written informed consent was obtained from participating police officers on the day of study participation. Interviews ranged from 40 to 120 minutes in length, were audio-recorded, and were transcribed verbatim. Interviews covered topics ranging from police duties to work–life balance, for example, participants were asked questions such as: “Could you please describe to me one of your typical working days?” and “Could you please describe to me a typical day where you must balance work and home life?”. 

### 2.2. Participants 

Participants (*n* = 17) were comprised of 9 women and 8 men. Most participants were white (88 percent), had completed some post-secondary education (29 percent) or a University degree (47 percent), and were married (58 percent) or dating/cohabitating (24 percent). Participants were aged between 31 and 59 years of age and were 44 years old on average. Most participants identified as heterosexual (94 percent) or gay (5 percent). Participants were constables (58 percent), followed by sergeants (24 percent), corporals (12 percent), and inspectors (6 percent), and most had an income above $100,000 before taxes (94 percent). Participants’ policing experience ranged from 5 to 26 years, and they had been working in policing for 15 years on average. There were no participants who identified as transgender, Two-Spirit, or gender non-conforming.

### 2.3. Qualitative Analyses

Interviews were recorded and transcribed verbatim. To prevent the early interviews from overly influencing the later ones, coding commenced only after the primary researcher believed the point of saturation had been reached (*n* = 17); i.e., no new information was observed by the researcher in the interviews. Field notes were taken throughout the interview process and helped in the observation of data saturation. The possibility of reaching a saturation point at 17 participants, particularly considering the relatively homogeneous population of Saskatchewan police officers, is supported by existing research on the concept of saturation [38]. Coding was done using a flexible deductive approach, supported by the qualitative data processing program NVivo 12 [39,40]. A sensitizing theoretical framework was used to deductively inform coding (i.e., using the literature to guide the identification of codes), with room for emergent and novel codes. For example, as the literature had demonstrated there were gender differences in work–life balance among men and women police officers, this finding was reflected in the coding. Codes were not created a priori; rather, the researcher was guided by concepts highlighted throughout the literature and the quantitative results. The coding process resulted in a total of 23 codes, further demonstrating the extent of saturation [38].

A post-positivist approach was used, therefore the research focused mainly on providing explanations, linking cause and effect, and relied heavily on the researcher’s critical examination [41]. An experienced qualitative researcher and coder reviewed the coding and provided written feedback. Thematic networks were created to represent, organize, and structure the data, allowing for analysis of themes individually and in relation to one another [39,42]. A focus on latent themes was favored, since the work of an officer is entrenched in police culture. Latent thematic analysis examined underlying ideologies and conceptualizations (e.g., community policing being perceived as women’s work and not “real” police work might represent the latent theme of hegemonic masculinity) [35,39]. 

## 3. Results

Several themes related to gendered experiences of police work were identified. A summary of the themes and their frequency, as well as an exemplar quotation, can be found in Table 1. Men and women readily identified gendered nuances and distinctions in how they experienced policing. Hegemonic masculinity appeared to be a common thread that pervasively impacted all officers through the police culture. Men and women both appeared to experience the repercussions of restrictive gender norms. All women indicated having experienced some form of gendered discrimination and/or sexual harassment, whereas no men reported having experienced or witnessed gender-based discrimination or harassment.

### 3.1. Internal Discrimination

The women who were interviewed reported experiencing specifically gender-based discrimination from their peers and their colleagues. Some of the discrimination was blatant and stated outright; however, women also experienced covert and subtle forms of gender-focused discrimination. The onus of accepting sexism appears to be placed entirely on women police officers, rather than the onus being on men to not perpetuate sexism. For example, a woman participant recalled a discussion on sexism with a superior officer:

*When I first got hired, I had one of my sergeants tell me […] he said: “Just so you know, I’m a sexist, like don’t be offended by it, but I’m a sexist”.*

(Woman 4)

Participants reported that officers routinely assessed women police officers as being less competent than men police officers due to their gender. The “old boy’s club” (Woman 2) resulted in an implicit bias, in terms of assessments from superiors. Specifically, women participants reported a double standard regarding competency ratings for men and women police officers, which led to women feeling the need to prove themselves more than their men counterparts:

*So, I mean in terms of like, the promotions…like there’s always someone who has to decide whether you get the job or not, right. Like even the promotion level, you’ve got like your test that you write which, that’s black and white, but then you’ve got your assessment from your sergeant and you have your assessment from your inspector. So all of those things, its all, you know it’s all biased in a sense right? Whether they like you or don’t like you, or you know. […] It’s harder for women because there’s a fine line between being assertive and then everyone thinking that you’re a bitch and that kinda thing. So does that affect your future stuff? Probably.*

(Woman 5)

Participants reported negative responses when a woman joined a shift or advanced in a position, with women being considered unfit for their roles. Women reported being undermined, less respected, criticized, judged more harshly, and treated unequally regarding their ability to perform occupational duties. Women participants also reported moving up the ranks at a slower rate than their colleagues who were men, despite outperforming them on relevant tests and tasks. Certain women choose not to pursue a higher rank position due to having experienced discrimination in the past. For example, participants reported that men were preferred over women for certain tasks despite comparable skill levels, which led women to becoming resigned to and expecting such experiences:

*So a supervisor who calls units to come to the call, but the first three people that show up are female, and then when the next group of people show up, they’re all male, he sends the females on their way, right. So those, I remember those standing out to me like: “I can’t believe this is even happening, we’re all trained and I’m actually a better shot than that guy.” And like, you just start thinking of things and then that creates that self-doubt, right. And so then a lot of us are stubborn and will push back and prove, but I feel like there’s more onus on us to prove our ability in the job, to stay in shape, to be a good shooter, to have like all that training under your belt.*

(Woman 2)

Women mentioned that there were noticeable differences in the types of calls and assignments that were attributed to men and women; however, women participants also reported their men colleagues were generally unaware of the distinctions. Woman 9 reported joining a new unit, yet experiencing a contradictory welcome from her superior officer:

*My […] boss’s boss of the unit at the time, kind of welcomed me to the unit but it was a really like hypocritical like: “Welcome to the unit, but [emphasis] I don’t think you should be here. And want you to get as much opportunity as possible but [emphasis] take all the vacation you want and like if you need to go and like want to go on vacation, go ahead”. Like it was kind of like a conflicting, like, glad you’re here, but not really kind of thing […] had that been a man at the same point, I don’t think anyone would have said anything, do you know what I mean?*

(Woman 9)

Women consistently reported that although objective measures were designed and implemented to provide men and women with the same opportunities, covert bias continued to exist and was facilitated by cohesiveness among men:

*A male’s idea or opinion could come across as more important that a female’s. So when a male speaks about something, they’re listened to. And a lot of times when a female speaks […] it’s overlooked or it’s like: “yeah, okay it’s a good idea but we’re not doing that”. So and because the men, men hang out together […] so they’re gonna listen to each other because they’re used to each other, so…*

(Woman 8)

### 3.2. Sexual Harassment

Women participants also described experiences of sexual harassment. This harassment from colleagues or superiors took many forms, ranging from activities categorized as “dark humor that went too far” (Man 2) to unwanted sexual contact. Women exclusively reported their difficulty in creating a reputation for themselves, which could easily be tarnished with rumors of affairs and sexual jokes that would lead to them being ostracized by their colleagues. Reports from women included feeling unsafe with a colleague or a superior officer, being groped between their legs in a patrol car by their training officer, being sent pornographic images, receiving a message from a colleague mentioning that he was masturbating while thinking of her, and having sexual objects (i.e., sex-toys) put in their workspace by their colleagues. All women participants had either directly experienced, or knew of a woman officer who had directly experienced, some form of sexual harassment. Some women participants reported choosing not to report these experiences in order to avoid anticipated career impacts. Other women participants reported pursuing formal routes of reporting sexual harassment and subsequently experiencing psychological trauma by reliving what happened by having to repeatedly recount the experience. Woman 2 reported realizing many of her colleagues had experienced some form of sexual harassment:

*I think all of our women are a community within themselves because there’s often discussions in the locker room about like, “I can’t believe that just happened,” or “I can’t believe that person said that to me,” and they’ll say, “That happened to me too.”*

(Woman 2)

One participant experienced harassment from her superior at the beginning of her career that led to her feeling “scared to do anything” and she describes this as the “worst experience in her career” (Woman 1). Another participant recounts her experience, which went on for years, as the cause of serious repercussions to her physical and mental health. She reported that sexual advances and comments from men started in police college and continued within her police service. She reported being shocked at their advances:

*But then it was like, okay, just ignore them, ignore them, ignore them, ignore them. And then it’s like text messaging, comments. And it’s, I don’t know, covert? It’s like, you know how it starts out, right? And then it would get to like some inappropriate comments, like even of superiors as you get into the organization. […] And it was for me shocking and inappropriate. And again we police but yet, allow that behavior?*

(Woman 6)

Most women participants had advised other women to be cautious of what they called “predators”. New women recruits are often sought out by men police officers in the police services, whether the women are married or not, and the advice from more senior women was to be careful of advances from certain men police officers. One woman had received advice specific for women during police college:

*When I was in police college, we had an inspector, who’s a female, come and talk to just the females. And she gave us this speech and she basically said: “Women in policing, get three titles, you’re either a bitch, a slut, or you’re gay.” And so she’s like, “kind of choose what titles you’re going to have. Because those are going to be your three titles”. And I always thought, I’m like really? Like that’s, like, kind of disheartening and like, gross. But as I went through it, I’m like, that’s very true.*

(Woman 7)

Women were specifically labeled in relation to how they engaged with men they were working with. On the one hand, she explained that a woman who did not engage in sexual relations with the men in her organizations was labeled as “a bitch” if she was not gay. On the other hand, a woman who did engage in sexual relations with a man colleague was then labeled “a slut”.

### 3.3. Motherhood and Parental Leave

Women described difficulty getting the same type of career opportunities as men due to disproportionate responsibilities for their family and as mothers. Men and women participants generally agreed that the societal and cultural expectations placed on women were also present in policing:

*So she [spouse of participant] basically put off her career to be able to get [child of participant] old enough that he could kinda look after himself sometimes. So I think that she made that sacrifice for sure and I think that would be pretty common for a lot of women in policing.*

(Man 3)

Mothers reported feeling more challenged to thrive both in their home lives and at work. For some, pregnancy resulted in being moved to a light duty section, impacting their colleagues or their unit, and producing feelings of being shamed (e.g., “oh well she is on maternity leave again” (Woman 5)). Women who choose to pursue senior positions and then go on maternity leave were reportedly judged to be “taking the opportunity away” (Woman 9) from someone more deserving. After a few children and a few years on light duty, women participants reported experiencing a negative connotation when returning to general patrol, feeling that certain colleagues were hoping such women police officers would not join their shifts. 

Relatively few opportunities for non-shiftwork positions exist. Women may be criticized for gravitating towards non-shiftwork to facilitate their roles as mothers. Men and women participants also reported that women in general, but mothers specifically, have more responsibilities at home, are more cognizant of the time away from their children, and spend less time on leisure activities than men. Accordingly, some women chose to wait until their children were older before joining policing and described the sacrifice of putting their careers on hold until they could fully commit to the occupation. There also remained senior officers who viewed motherhood unfavorably, as illustrated by the interaction Woman 1 reported with a chief from a different police service:

*But hearing the Chief of Police from one of the police agencies make a comment that, when we were talking about diversity how […] it’s very difficult to hire women because women don’t want to be hired because they want to have babies. And the women they do hire impacts the police service because they take maternity leave. I couldn’t be quiet when they said that so I had to stick up my hand and address that […] but the fact that we still have police officers thinking that says there’s gender inequalities.*

(Woman 1)

There were also important challenges for men who wanted to start a family due to the outlook of some police services on parental leave. Men could advance in their careers at rates women (who were often the primary caretakers) could not; this appeared to come at the cost of spending time with their family:

*Whereas male police officers, I mean we just go in and work, right. And we don’t have any benefits for paternal leave we just go straight to EI so it’s, we’re not encouraged to take time off, it’s more discouraged, as much as it can be discouraged I guess without becoming illegal or becoming, so… we’re just, you go in and work and the harder you work the harder you move so we have a little more opportunities.*

(Man 8)

Another challenge mothers face is the financial strain that having children imposes in terms of pension and benefits. Women mentioned that since they did not have the option to opt out of pension plans and benefits, some women felt that they could not afford having another child.

### 3.4. Identity

Differences emerged in terms of how participating men and women police officers described their identities. During the interviews officers were invited to share any information they would like to share about themselves with the interviewer. Most of the men answered the question in terms of their careers (e.g., length, positions held, rank). Women endorsed policing as part of their identity, but only as a part of a larger identity that included many different roles (e.g., sister, spouse, mother, stepmother, daughter) and accomplishments outside policing (e.g., degrees, previous careers, recreational activities). All participants consistently described the difficulty of occupational demands requiring them to set aside their identity as an officer when off-duty; however, there appeared to be a distinction between men and women, particularly for older men who had “lived and breathed policing” (Woman 3), and who experience a loss of identity after retiring from policing. Woman 5 reported the differences between her and her spouse, both of whom were police officers, in how they identified themselves in relation to policing:

*Where my husband’s more like […] defined in a way by being a police officer, like that’s his life. And there’s a lot of guys at work who are like that, too. Whereas I’m like, you know, yeah this is what I do for a living, like as a job, but I had a whole life before I started this job. I went to University, I have a degree […] I have a diploma, I have friends.*

(Woman 5)

The identities of men and women police officers may differ based on the factors that led them to careers in policing. Men police officers mentioned the importance of their oath and how their oath made fully letting go of their officer role difficult, even when off duty. Some men reported there was no “big shift” (Man 5) for them between on and off duty, as their personalities are entrenched in their role of being an officer. Some men police officers reported it taking years for them to learn how to let go of policing as their primary identity. Others reported that leaving the area where they work, for example, going on vacation or living somewhere remote, helped them let go of their primary police identity; however, men participants also reported being “held to a different standard” (Man 1) as police officers relative to civilians, perceiving being “expected to act a certain way and do certain things” (Man 3). Therefore, the perception of being different from civilians and as having different occupational expectations than women police officers may further strengthen the primary identity of being a man police officer, over and above anything else. 

Participants reported that police officers who were at the intersection of identities such as gender, race, and sexual orientation, experienced a greater level of hardships. Due to the sample size, and in order to protect the confidentiality of the police officers interviewed, details regarding each participant will be omitted. Intersectionality in policing is particularly challenging because officers already stand out as a function of their vocation and uniform, which may exacerbate prejudices related to gender, race, and sexual orientation. Participants of different cultural backgrounds reported experiencing a “little more discrimination and racism on the street” and “racism” in the “public” and in the “organization”, which is due in part to working in a province where the general population and police services are predominantly white. Participants at different intersections reported “feeling like you’re alone” and anticipating a shorter career longevity for officers who are outside of the normative cis-gender, white, male officer, unless they have a “strong advocate and a strong support person”. A participating officer explained that minority officers may leave policing earlier due to “the way we are treated and the inequities. Absolutely the inequities.” 

One participating officer found it “a little scary” to join policing due to their non- normative identity, but then were surprised at the acceptance they received. People were “curious”, but the officer reported feeling respected by their organization and the public. The officer described their “work ethic” as a factor in being “accepted”, which suggests officers who do not fit the contemporary archetype for a police officer may experience internal or external pressure to prove themselves in order to be successful.

### 3.5. Stereotypically Feminine Attributes

Police participants reported that women police officers possessed stereotypically feminine attributes, which they believed impacted how women policed. Many participants reported that a woman’s presence or her way of policing could have significant occupational advantages. Women were described as having a calming presence on both civilians and their fellow officers. Women were also described as having different skillsets to men, which often became assets in their careers. Stereotypically feminine characteristics ascribed to women police officers included being nurturing, communicators, softer, listeners, motherly, gentle, and analytical. Some men indicated that feminine qualities made women better officers and better occupational partners because they were able to bring balance to their work and their partnership.

*Typically, our bad guys are male and to have that female presence, whatever reason it calms them down. You can have a proper conversation […] having that female presence definitely brings everything down a little more and, I, I feel like females are great communicators compared to men, they listen better, they talk better, they’ll respond better to situations, than some police officers that are male that can do that.*

(Man 2)

Participants also reported women police officers were expected to take on more of the emotional labor due to their kindness and thoughtfulness. Woman 2 recalled being partnered with men due to an observed ability of women to provide a calming presence; nevertheless, she preferred working alongside another woman:

*And so, it’s kind of interesting how throughout my patrol years, certain people were placed to work with me because I was female. And so because I was patient, calm, kind of a different personality than other people than they would marry us together to try and even it. And my best years of patrol were when I’ve worked with another female partner, because we had very little altercation and confrontation with people because we were both understanding, patient, didn’t look intimidating.*

(Woman 2)

Some men also described their preference for being partnered with a woman:

*You know men and women police differently. I think men will make a lot of decisions very, very quickly and that processing time sometime has to happen. Even though in a high stress situation sometimes you don’t have that time. I found women process things a little bit differently, especially when they’re newer to the job. They process a little bit differently. They make less snap decisions. They’re not as willing to go to a physical sort of confrontation, which is good. Generally, I find them easier to work with. I work way better with women than I do with men. And I like the balance that we have.*

(Man 3)

Stereotypically feminine qualities were also a distinguishing element between men and women police officers with respect to promotion. Participants described what would make a woman stand out in a high rank, emphasizing work–life balance, respect, worth, and fairness. Participants also described women who stand out as possessing some stereotypically masculine qualities (e.g., “they’re at the gun calls […], tough […] corrected the lazy asses’ behavior” (Woman 3)), illustrating that women need to possess both sets of qualities (i.e., “cover all of those bases” (Woman 3)) to be promoted. 

Stereotypically feminine qualities did not always have positive connotations. Women were described as being more social and talkative, which was sometimes described as a nuisance that negatively impacts task completion, and some men also reported feeling women used the “female card” and their gender to get out of work tasks. Some participating men police officers reported concerns that women policed incorrectly more than men because of differences in “compassion”:

*I can really see it on calls, where [women] gravitate to the victim, if they can go help the victim and take a statement from them, the male officer will make an arrest. Like I’ve been to a house where there was three or four of us there and before we cleared the apartment, she went and started taking a statement from the victim […] that’s a big no-no for saving our backs and stuff like that. I can kind of see, our females and like I said, I know other departments, there are some good females, but ours are more compassionate for victims or even there’s, the suspects, circumstances, right, “Oh that’s the way they were brought up” and stuff like that. Well, you’re still making your own choices, you have free will, right?*

(Man 5)

### 3.6. Hegemonic Masculinity

Overall, masculinity appeared to be highly valued and was described as a central tenet of policing. Participating men police officers indicated that through policing they developed that “tough mentality personality” (Man 2), with a push to be “more rigid, follow order, and leave emotions out of it” (Man 5), being “almost primitive” (Man 8). Men participants reported feeling, and feeling expected by others, a need to be big, strong, brass, and tough in order to “catch bad guys” (Man 4) and “fight” (Man 6). Man 4 reported no recall of ever witnessing a fight between a civilian and a woman officer, but added that, for him, a fight is “actually punching, blood flying, that’s a fight”, implying men were more likely to engage in violent altercations than women. 

Feminine qualities may be seen positively, but appear to remain outside of policing norms. As part of hegemonic masculinity, expressing emotions is seen as feminine, not valued, and tolerated more for women than men. Women participants reported their emotions were ascribed to their gender and reported not being taken seriously or being dismissed for displays of emotion (e.g., being upset or crying). Man 5 reported that men police officers were perceived as “assholes” rather than “lovey-dovey”. Police officers may present an image of being unemotional; however, participating men and women comparably reported experiencing emotional challenges related to policing. Men participants also reported that having a woman partner instead of a man facilitated more discussion about thoughts and feelings following calls for service.

*That we as police, we never deal with our own feelings, because we’re hard, we’re tough, we don’t cry, which is, which is total bullshit. I’m sure there’s probably enough members out there that are crying but we just never see it.*

(Man 6)

Women’s expression of emotion may be tolerated based on gender expectations, but the devaluation of feminine traits may lead women to reject their emotions to conform with the masculine gender norms associated with policing:

*And I think as a female, certainly back then, in that male-dominant of “suck it up, buttercup” kinda thing, you just think as a female: “Oh I’m just being overly emotional…”*

(Woman 3)

Women police officers ascribed masculine traits to their men colleagues. For example, Woman 1 mentioned that men were “enforcers” who “liked the power” their occupation afforded them, and that they tended to “shake things off a little more” than women, further emphasizing restrictive gender norms. To achieve a higher status, men police officers strived to be as manly as possible (e.g., “SWAT just seems like this ultimate male goal to prove how manly and how awesome you are” (Woman 5)). Taking too many precautions was also seen as feminine, as Woman 9 recalled being ridiculed by the men working with her for wearing her protective gear at a gun call; in contrast, participants also described a hierarchy among the men based on the masculinity of their presentation. Men were reported to be demoted in status, falling down the hierarchy, for demonstrating more stereotypically feminine qualities:

*[Police culture] might even be tougher on, if you’re not a stereotypical male, if you’re a softer, gentler, smaller male. I think you might be more, I know I’ve seen it, impacted more harsh, than that alpha male. I think the women generally we can hang in the middle pretty good. And we can hang out with the big dogs or we can hang out with the not-alpha-males. But I’m gonna say the non-alpha males are picked on bad.*

(Woman 3)

The central role of hegemonic masculinity in policing appears to favor both women and men who display traits associated with highly valued forms of masculinity. Women 3 described being treated very well by her colleagues and reported that she joined policing as “a very athletic and capable and a strong woman”. The differences in how women are perceived appears poignant for women who are physically stronger or more confrontational (women who are “absolute tigers, not afraid of anything” and therefore “great to work with” (Man 2)); nevertheless, women mentioned that there was a fine line between possessing these qualities and being too assertive (i.e., “labeled as a bitch” (Woman 5)).

## 4. Discussion

The current research provided a detailed presentation of the gendered experiences of participating police officers. Narratives from women and men police officers can facilitate an understanding of how gender may impact their occupational experiences and treatment. The results have potential implications for policies and programs to assess policing with a gendered lens, to identify and reduce gender-related biases, and to promote the mental well-being of all officers. 

Participating men and women police officers frequently described policing as a male-dominated occupation, in which there was frequent and impactful internal (i.e., organizational) sexism. Participating women police officers described being excluded from some call types (e.g., frontline team at a domestic call), but repeatedly selected for others (e.g., cases of sexual assaults), and having learned through experience to expect gendered distinctions of police duties. The current results are consistent with previous research evidencing that some commanding officers attempt to gender-match officers to specific calls that required skills typically attributed to either women (e.g., listening) or men (e.g., physical strength); however, the sex-based selection bias appears specific to women police officers [21]. 

Gendered classification of skills ultimately strengthens the dominance of men within policing by limiting women to certain types of duties. Researchers have argued that gendered assignment of duties is another form of tokenism [43]. The “token” woman officer is preferred over men when dealing with cases involving sexual assaults and women civilians, leading women police officers to feel they are perceived more in terms of their gender than in terms of their competency and individual skills [43]. The conflict between work identity and gender identity may be lessened for women when units are gender-balanced, in which women report feeling less devalued [44]. Men and women in the current sample reported positive experiences when working in gender-balanced partnerships (e.g., on patrol). 

Women police officers must balance gender norms, stereotypes, and police work simultaneously [20], which requires additional emotional labor beyond what is expected of men [45,46,47]. Emotional labor involves the regulation of emotions, both for the self and others, and is commonly associated with feminized service and “front line” professions. In policing, emotional labor may be elicited by both the nature of the duties assigned to female officers (e.g., support of victims) and the ongoing negotiation of their gender performance to manage the emotions of others while navigating sexism and stereotypes. Women police officers may engage in additional emotional labor with their colleagues (e.g., through formal or informal peer support), because doing so is consistent with contemporary gender norms; specifically, women function more often as caregivers [48], and men in the current sample appeared to feel more at ease when expressing emotions in front of women, perhaps because this did not jeopardize their social standing with other men. To date, very little research has examined gender and emotional labor in policing [49]. The expectations placed on police women to engage in more gendered and stereotypically feminine tasks (e.g., responding to sexual assaults, engaging in emotional labor) appears to reflect the societal trend found at a broader organizational level, where women’s roles and duties are gendered through benevolent and hostile sexism [50].

Many officers described what they termed police dark humor, which was described as sometimes having gone too far. The humor was often sexual, denigrating, or gendered when directed towards women. Human resources departments were described as supportive towards the recruitment and retention of women, but participants reported a large gap between formal human resources protocols and the less formal internal culture. Some participating women also reported opting not to pursue an official complaint to their human resources department because the process was too distressing or because they feared career repercussions. The current results are consistent with previous reports of sexual harassment in policing [7]. Internationally, three out of four women working as police officers experience sexual harassment [51]; however, women in policing experiencing more sexual harassment than women in other occupations has not resulted in more formal complaints [52]. Researchers posit that, as women are integrated in police work, internal resistance to their integration may maintain and even increase sexual harassment [52]. Women participants in the current sample described sexual harassment as having psychological and physical repercussions, which helps explain previous associations between sexual harassment and sick leave in women police officers [9]. 

The glorification of an idealized version of manhood (i.e., hegemonic masculinity) [53] was consistently described as a concern by participating men and women. Participants equated characteristics of policing with traits contemporarily viewed as masculine such as stoicism, violence, strength, size, and control; conversely, participants also underscored the utility of traits contemporarily viewed as feminine for policing, such as empathy, calmness, communication, and vulnerability, which were associated with femininity. Participating men described police as valuing feminine traits, but only when the traits were expressed by women. The stereotypical masculine archetype of a police officer appeared pervasive for participants, despite recognizing the benefits of stereotypically feminine strengths for officer safety and promoting positive public relations. The paradoxical engagement with gendered traits highlights challenges with adhering to rigid socially constructed notions of gender for policing as an occupation; indeed, structural gender-based polarization in policing (e.g., men manage SWAT, women manage sexual assault) may eliminate important pervasive benefits and create areas of potentially problematic hypermasculinity [54]. 

Women reported experiencing many negative consequences of hegemonic masculinity (e.g., gendered discrimination, harassment, sexism); relatedly, men also reported experiencing many negative consequences from hegemonic masculinity (e.g., not discussing feelings related to challenging calls or experiences). Theories of hegemonic masculinity postulate that men are placed on a hierarchy based on how well they fit certain hypermasculine traits (e.g., physical strength) [31,53]. Hegemonic masculinity places women in an entirely different category from men, but men are tasked with competing to achieve ever-higher positions in a masculine hierarchy through occupational (e.g., SWAT team, firearm instructor) and other achievements (e.g., physical strength, sexual conquests). Among police officers, lower social status has been associated with greater chronic stress [55]. The sense of belonging to a group and a community (us versus them) is a core tenet of policing and hegemonic masculinity [56]. Accordingly, women as outsiders may rely on their own group and men who fall short of the hypermasculine traits may be even more ostracized [53]. The impact of hegemonic masculinity on police culture created pressure on men police officers to appear brave and in control, leading to potentially problematic emotional suppression [57]. Participating men reported experiencing more freedom to express their emotions and discuss the harsh realities of policing with their women colleagues, reiterating the significance of women’s emotional labor.

There were several study limitations that provide directions for future research. First, officers in the current sample self-selected for participation in the survey and in the interviews. The main research objectives of better understanding the role of gender on police mental health was stated on recruitment materials. Therefore, officers who are particularly aware of, or concerned about, gender differences may have expressed greater willingness to participate. Second, while the current analyses compared gender, it should be noted that due to the large overlap between sex and gender in the current sample, it is difficult to fully discriminate between one or the other. In fact, West and Zimmerman conceptualize gender as “doing”; acting according to societal norms, pressures, and expectations placed upon one’s gender, and sex as “being”; particularly based upon the recognition of one’s physical sex [58]. Therefore, certain elements mentioned by participants, such as the recognition of a female body as being less powerful than a males’, encompasses both the “being” and the “doing” entrenched in police officers’ experiences. The interlaced nature of sex and gender may also impact the current results and warrants additional focus in future research. Third, the current interviews were conducted with officers of various ranks and experiences throughout Saskatchewan, but the results may not be generalizable to other provinces or other centers. Future research should attempt to gain a better understanding of women and men police officers’ mental health across Canada, including assessing for differences based on the population density of services areas (e.g., urban relative to rural policing environments). Finally, the current results are exploratory in nature. Evidence suggests that while both men and women police officers are at a greater risk of mental disorders than the general population, the risk is increased for police women, relative to men [5,6]. While the current research highlights important differences in the experience of policing for men and women officers, more research is needed in order to understand how a career in policing, coupled with experiences of gender-discrimination, specifically contribute to women police officers’ mental health challenges. 

## 5. Conclusions

The current study produced a more in-depth understanding of differences in the reality experienced by men and women police officers and how their experiences may relate to or impact mental disorder prevalence. Police officers identified several initiatives which could help support police officers through their occupational challenges and improve their physical and mental well-being. Women participants provided concrete solutions to help support a healthy and supportive work–life balance, wherein women would not feel remorse for having a child; for example, increased staffing, formal succession planning in advance, and having on-site daycare. Many women participants reported that an on-site daycare would reassure them that their children were properly cared for while working difficult hours, such as night shifts. Participating officers indicated that daycare would benefit a number of working parents that experience shiftwork (e.g., nurses). Pension plans and benefits appeared to further burden women officers during their parental leave. Involving women, not only in higher ranking positions, but in the creation and implementation of policies would ensure that their realities are considered. 

Participating police officers did not offer specific recommendations regarding the sexism and discrimination the women experienced; nevertheless, there was an apparent need for supportive and preventative measures. Men police officers appeared to be aware of gender-based challenges for their women counterparts in regard to work–life balance and interactions with the public; however, men were less likely to report the sexual harassment and gender-based discrimination women reported experiencing from their colleagues and superiors. As police services attempt to become more inclusive, challenges pertaining to the gendered reality of police work as experienced by women need to be systematically resolved [59]. Increasing knowledge and awareness among men of the sexualization and dismissal of women police officers is an important first step in ensuring women have a fair and supportive work environment. Women police officers reported being mentored, as early as during police college, to expect gender-based discrimination, which is particularly disappointing given the important role police officers play in reducing gender-based discrimination among civilians. Police services should strive to actively teach men about the gendered challenges women experience and to encourage men to actively and collaboratively improve the work environment for women by reducing discrimination, increasing safety, and achieving real parity.

## Figures and Tables

**Table 1:** Summary of themes and their frequency.

Theme	Number of Participants	Number of References	Quotation
Discrimination	11	51	“*It’s very routine to have those very noticeable—the men don’t notice it—but the women do […] the men will predominantly be called first on the high-risk calls and the women will be dispatched to, even to this day, the old lady who needs comforting for something.*” Woman 5
Sexual Harassment	8	48	“*If you’re the new girl, there’s always an assumption of rumors of who you’re sleeping with. I had lots of people come to me inappropriately.*” Woman 2
Motherhood and Parental Leave	12	24	“*I know that most police services want women in there, they want them to have families, careers, they say they want this work–life balance but then if women get pregnant and have families, it’s not really made that easy for them. It’s not a priority within the service to support that and it’s just, it feels more, and then obviously just going through it, it feels more like it’s a pain and they will allow you to do it, but they don’t really want you to.*” Man 8
Identity	17	31	“*Not totally [on being able to let go of policing on days off], I take my oath very seriously and I know a lot of other police officers do.*” Man 2
Stereotypically Feminine Attributes	16	52	“*I still think that women expect us to be more hands on. […]men are more likely to be hands-on physical and women would rather use the tools that they have.*” Man 3
Hegemonic Masculinity	17	85	“*It’s still almost a primitive world out there sometimes, where it’s very physical with men, people will try to engage more with women and talk to them.*” Man 4

## Data Availability

The data sets generated and/or analyzed during the current study are not publicly available due to guarantees made in the data collection consent form regarding protections to ensure participant confidentiality.
